# Lipoprotein Subfractions in Metabolic Syndrome and Obesity: Clinical Significance and Therapeutic Approaches

**DOI:** 10.3390/nu5030928

**Published:** 2013-03-18

**Authors:** Dragana Nikolic, Niki Katsiki, Giuseppe Montalto, Esma R. Isenovic, Dimitri P. Mikhailidis, Manfredi Rizzo

**Affiliations:** 1 Biomedical Department of Internal Medicine and Medical Specialties, University of Palermo, Palermo, 90127, Italy; E-Mails: draggana.nikolic@gmail.com (D.N.); giuseppe.montalto@unipa.it (G.M.); 2 Second Propedeutic Department of Internal Medicine, Medical School, Aristotle University of Thessaloniki, Hippokration Hospital, Thessaloniki, 54124, Greece; E-Mail: nikikatsiki@hotmail.com; 3 Laboratory of Radiobiology and Molecular Genetics, Institute Vinca, University of Belgrade, Belgrade, 11000, Serbia; E-Mail: isenovic@yahoo.com; 4 Department of Clinical Biochemistry (Vascular Disease Prevention Clinics), Royal Free Campus, University College London Medical School, University College London (UCL), Pond Street, London, NW3 2QG, UK; E-Mail: mikhailidis@aol.com; 5 Euro-Mediterranean Institute of Science and Technology, Palermo, 90139, Italy

**Keywords:** lipoproteins, small dense low density lipoprotein, obesity, metabolic syndrome, obesity treatment, anti-obesity drugs, lipid-lowering drugs

## Abstract

Small, dense low density lipoprotein (sdLDL) represents an emerging cardiovascular risk factor, since these particles can be associated with cardiovascular disease (CVD) independently of established risk factors, including plasma lipids. Obese subjects frequently have atherogenic dyslipidaemia, including elevated sdLDL levels, in addition to elevated triglycerides (TG), very low density lipoprotein (VLDL) and apolipoprotein-B, as well as decreased high density lipoprotein cholesterol (HDL-C) levels. Obesity-related co-morbidities, such as metabolic syndrome (MetS) are also characterized by dyslipidaemia. Therefore, agents that favourably modulate LDL subclasses may be of clinical value in these subjects. Statins are the lipid-lowering drug of choice. Also, anti-obesity and lipid lowering drugs other than statins could be useful in these patients. However, the effects of anti-obesity drugs on CVD risk factors remain unclear. We review the clinical significance of sdLDL in being overweight and obesity, as well as the efficacy of anti-obesity drugs on LDL subfractions in these individuals; a short comment on HDL subclasses is also included. Our literature search was based on PubMed and Scopus listings. Further research is required to fully explore both the significance of sdLDL and the efficacy of anti-obesity drugs on LDL subfractions in being overweight, obesity and MetS. Improving the lipoprotein profile in these patients may represent an efficient approach for reducing cardiovascular risk.

## 1. Introduction

A sedentary lifestyle and high energy diet increases the prevalence of being overweight, obesity, metabolic disorders (e.g., metabolic syndrome, MetS) and type 2 diabetes mellitus (T2DM) [1]. Obesity and obesity-related co-morbidities, such as MetS and T2DM, are a major health problem worldwide [2]. Each of these disorders, in addition to established vascular risk factors (e.g., dyslipidaemia, smoking and hypertension) increases the risk of cardiovascular disease (CVD). Therefore, the American Diabetes Association and the American College of Cardiology Foundation recommend a multifactorial risk reduction strategy targeting each risk factor and underlining both lifestyle and pharmacological treatment [3].

A commonly observed lipid abnormality in obese individuals (especially those with excess central adipose tissue), is an increased presence of small dense low-density lipoprotein (sdLDL) [4,5,6]. This predominance of sdLDL particles is associated with raised triglycerides (TG) and decreased high density lipoprotein cholesterol (HDL-C) levels, forming the so-called “atherogenic lipid triad” [7,8]. Adipose tissue also produces a diversity of adipokines that play a role in the pathogenesis of inflammation, dyslipidaemia and hypertension [9] that increase the rate of CVD morbidity and mortality linked to obesity, T2DM and the MetS [10]. Excessive visceral adiposity enhances the availability of free fatty acids (FFA) that lead to TG accumulation in muscle and liver (fatty liver) and increase circulating TG levels, due to the enhanced hepatic production of very low density lipoprotein (VLDL) cholesterol [11,12,13]. Furthermore, the raised flux of FFA and TG to muscle and other tissues induces insulin resistance (IR) [14,15].

LDL varies in size, density and metabolic characteristics and comprises at least four distinct subclasses (large LDL-I, medium LDL-II, small LDL-III and very small LDL-IV (Table 1)), with sdLDL being associated with increased CVD risk [16]. Although the association between IR and increased LDL-C levels is not typical, elevated sdLDL levels with lower large LDL concentrations are associated with reduced insulin sensitivity and increased adiposity [17,18,19]. Generally, two phenotypes have been described: pattern A, with a higher proportion of larger, more buoyant or medium-sized LDL, and pattern B, with a predominance of sdLDL [16]. The difference in size and density among LDL subclasses is due to variations in surface lipid content and conformational changes in apoB-100, including increased exposure on the particle surface [20].

**Table 1:** Physicochemical properties of low-density lipoprotein (LDL) subclasses (adapted from [11]).

		Peak S_f_	Density Peak	Diameter (Å)	Protein	Cholesteryl ester (%)	Unesterified cholesterol (%)	Triglycerides	Phospholipids
			(gm/mL)		(%)			(%)	(%)
***Pattern A***	***large LDL-1***	7–12	1.019–1.023	272–285	18	43	9	7	22
	***medium LDL-2***	5–7	***a*** 1.023–1.028	265–272	19	45	10	4	23
			***b*** 1.028–1.034	256–265	21	45	9	3	22
***Pattern B***	***small LDL-3***	3–5	***a*** 1.034–1.041	247–256	22	46	8	3	21
			***b*** 1.041–1.044	242–247	24	44	7	3	21
	***very small LDL-4***	0–3	***a*** 1.044–1.051	233–242	26	42	7	5	19
			***b*** 1.051–1.06	220–233	29	40	7	6	18

S_f_: Svedberg flotation.

*Search Strategy*: We searched PubMed and Scopus listings for relevant publications using combinations of the following keywords: “lipids”, “lipoproteins”, “small dense low density lipoprotein”, “high density lipoprotein”, “overweight”, “obesity”, “obesity treatment”, “anti-obesity drugs”, “lipid-lowering drugs” and “new anti-obesity drugs”.

## 2. The Clinical Relevance of sdLDL

sdLDL tend to coexist with elevated TG and low HDL-C levels and together comprise the “atherogenic dyslipidaemia” pattern, which appears to be heritable, but also several non-genetic factors, such as abdominal adiposity, influence the expression of this phenotype [16]. However, it is still debated whether LDL particle size is an independent CVD risk factor after adjustment for TG and HDL-C levels [21].

In relation to large buoyant LDL, sdLDL particles are taken up more easily by arterial tissue, show lower affinity for the LDL receptor, have a longer half-life in plasma and greater oxidative and glycation susceptibility, suggesting a link between sdLDL particles and atherogenesis [22,23]. It has been shown that subjects with high levels of sdLDL particles have an approximately three- to seven-fold increase in the risk of developing coronary heart disease (CHD), independently of LDL-C concentration [24,25,26]. Some studies found that subjects at high CVD risk, such as those with peripheral arterial disease or abdominal aortic aneurysm, may also have higher levels of these particles [27,28]. Further, a significant relationship between LDL size and the occurrence of carotid atherosclerosis was reported [29,30,31]. In different metabolic diseases (e.g., polycystic ovary syndrome, growth hormone (GH) deficiency) [32,33,34] and in women with gestational diabetes [35], increased levels of sdLDL were also found. 

sdLDL also represents a marker for diagnosis and severity of the MetS [36,37]. In this context, we confirmed that sdLDLs are increased in the MetS and showed an independent predictive role for future cardiovascular and cerebrovascular events [38]. Additionally, it has been suggested that the sdLDL-C/LDL-C ratio correlates with various parameters associated with MetS rather than the LDL-C or sdLDL-C levels, thus possibly representing a more useful clinical indicator [39]. Furthermore, an increased sdLDL-C/LDL-C ratio was an independent factor determining decreased adiponectin levels in MetS patients [39], but the mechanisms involved need to be elucidated.

## 3. Overweight, Obesity and sdLDL

Visceral obesity and IR have been recognized as the main causes of raised levels of sdLDL, because these factors contribute to postprandial hypertriglyceridemia; an essential mechanism is increased FFA release from adipocytes, which stimulates hepatic TG output [11,13]. Additionally, if fatty liver is present, upregulated *de novo* synthesis of FFA may increase hepatic TG production and affect the hepatic metabolism of TG and/or LDL-C, resulting in increased sdLDL levels and rapid atherogenesis in patients with MetS or T2DM [40]. In such metabolic conditions, fatty liver may enhance atherogenesis by raising the levels of sdLDL particles [41,42]. Hosoyamada *et al.* suggest that fatty liver may affect LDL particle size independently of both visceral obesity and IR [40]. Thus, treatment of fatty liver might decrease atherogenesis in MetS or T2DM by reducing sdLDL-C levels. 

Currently, there is renewed interest in the usefulness of measuring non-fasting TGs, as a more powerful and independent predictor of CVD risk than fasting levels [43]. In this context, an expert panel provided a consensus statement on the classification of non-fasting TGs concentration and other related clinical recommendations [44]. Standard reference values for post-prandial TG levels were suggested based on a recent meta-analysis [45].

Data from the Mima study [46] indicate that a polymorphism of the beta(3)-adrenergic receptor gene (the genetic marker for obesity-related traits) is correlated with the area percentage of sdLDL (*p* < 0.05), independently of age, gender, body mass index (BMI), smoking and IR index, suggesting a genetic predisposition to increased sdLDL in the presence of this polymorphism.

Weight loss in obese, non-diabetic women resulted in a significant reduction of the levels of lipoprotein-associated phospholipase A2 (Lp-PLA2) (*p* < 0.01), a phospholipase primarily associated with LDL, especially with sdLDL, while neither the cholesterol levels of sdLDL particles nor the percentage of the sdLDL-cholesterol of the total LDL-C were changed [47,48]. This change in Lp-PLA2 activity correlated with the changes in VLDL levels (*p* < 0.05) and could be a potentially new predictor for atherosclerotic disease. Lp-PLA2 is also a marker of sdLDL in human plasma [49].

Alternate day modified fasting (ADMF) is a dietary restriction that could help overweight and obese individuals to lose weight and lower CHD risk [50]. After eight weeks of ADMF, peak and integrated LDL particle size, as well as the proportion of large LDL particles increased, whereas the proportion of small and medium particles decreased (for all comparisons, *p* < 0.05). Cholesterol level in large particles did not alter, whereas this parameter was reduced within small- and medium-sized LDL particles (*p* < 0.05). Furthermore, LDL particle size was associated with reduced body weight (*p* = 0.04) and smaller waist circumference (*p* = 0.03). On the other hand, no association was observed between LDL particle size and plasma TGs (*p* = 0.11) or HDL-C concentrations (*p* = 0.65) [51], although reductions in plasma LDL and TGs concentrations were observed. However, given that the effect of weight loss on LDL particle size was not evaluated separately from the effect of fasting, it remains unclear whether these cardioprotective effects were due to reduced body weight or the prolonged fasting period. Future studies are needed to elucidate these effects on changes in lipoprotein subfractions.

The effects of glucose- or fructose-sweetened beverages have been investigated in overweight and obese subjects, and differential effects were shown on adipose distribution [52]. Both total abdominal fat and visceral adipose tissue (VAT) volume were increased in individuals consuming fructose, while only subcutaneous adipose tissue volume was significantly increased in those consuming glucose. Fructose raised sdLDL, oxidized LDL (oxLDL) and postprandial remnant-like particle lipoprotein-cholesterol (RLP-C) and RLP-TG, whereas glucose consumption did not [44]. Furthermore, sdLDL was mostly affected by pre-existing MetS risk factors (MSRF), and the increase in sdLDL levels during fructose consumption was more than two-fold greater in subjects with three MSRF than in those with zero to two MSRF [52]. Finally, consumption of fructose at 25% of energy requirements with an *ad libitum* diet decreased glucose tolerance and insulin sensitivity (greater in women than in men) compared with glucose consumption.

A high prevalence of sdLDL in obese children, as well as a relationship between peak LDL diameter and abdominal fat accumulation, and the level of both HDL-C and TG was reported [53]. When changes in LDL subfractions during a weight loss intervention for overweight and obese children were investigated, a reduction in cholesterol concentration of LDL III particles (54.1 *vs.* 40.4 mg/dL; *p* < 0.01), and shift in mean LDL-C peak particle density (1.041 *vs.* 1.035 g/mL) was found [54]. These alterations were also positively and significantly correlated to changes in VLDL metabolism. In contrast, in a later study [55], statistical difference was reported in the LDL particle size between 26 obese and 27 healthy children (*p* = 0.575); the size of LDL particles was not correlated with BMI, homeostasis model assessment (HOMA)-IR or serum lipids, although obese children had increased TGs and low HDL levels. Based on these results, the authors suggested that LDL particle size measurement is not necessary in childhood obesity as a routine procedure. A potentially explanation for these different results might be genetic factors and age in different populations [55]. Postprandial pro-atherogenic factors in obese boys were significantly improved after two different meals: moderate fat (MF: 61% carbohydrate, 27% fat) *vs.* high fat (HF: 37% carbohydrate, 52% fat). OxLDL concentration was raised after the HF, but not after the MF meal (9.3(2.2)% *vs.* 1.8(2.2)% from baseline, *p* < 0.02) and the densest LDL particles were associated positively (*p* < 0.05) with oxLDL levels. HDL-C concentration was lower (*p* < 0.05) at 300 min after HF *vs.* MF meal [56]. These results indicate that an evaluation of postprandial lipids may be relevant in children, as suggested earlier [57]. 

Reduced GH secretion in obesity could be associated with atherogenic dyslipidaemia, including raised sdLDL particles, given that reduced peak-stimulated GH in obesity was independently associated with a more atherogenic lipoprotein profile defined in terms of particle size (*p* < 0.0001) [58,59]. In this study, obese patients with reduced GH secretion had a smaller mean LDL and HDL particle size in comparison with normal weight or obese subjects with sufficient GH (*p* < 0.0001).

In another study where the ethnic difference in lipid levels and LDL particle size and subclasses were investigated, obese black women had significantly more sdLDL (subclass IV) compared with obese white women [60].

In summary, the entities of MetS and obesity, with a clinical clustering of CV risk factors and underlying IR, are also associated with phenotypical changes in lipoprotein patterns that increase the atherosclerotic potential of circulating lipids and their interaction with the vessel wall and endothelium. LDL subfraction is positively correlated with higher waist circumference, higher serum TG, as well as older age and more statin usage in hypertensive patients. Thus, LDL size could represent a risk factor associated with endothelial dysfunction in hypertension [61]. Further, *in vitro* studies, using cultured endothelial cells, have shown that LDL subfractions from hypercholesterolemic patients may deregulate endothelial function [62].

The main outcomes of discussed studies in this review are listed in Table 2.

**Table 2:** Outcomes of the observed studies that have analysed small dense low density lipoprotein cholesterol (sdLDL) in overweight and obese subjects.

*Study*	*Patients*	*sdLDL and measurement*	*Outcome of the study*
Satoh N *et al.* [39]	214 subjects (97 men and 117 women)	↑	The ratio of sd-LDL-C/LDL-C is a more useful clinical indicator than sdLDL
		in subjects with MetS A dual detection HPLC and a Lipoprint LDL system	
Tsuzaki K *et al.* [46]	277 rural Japanese subjects	↑	Genetic predisposition to increased sdLDL in subjects with polymorphism of the beta(3)-adrenergic receptor gene
		Lipoprint System (Quantimetrix, Redondo Beach, CA)	
Tzotzas T *et al.* [47]	28 obese, non-diabetic women	↔	A low-calorie diet reduces the levels of Lp-PLA2; the levels of sdLDL particles were not changed
		3% polyacrylamide gel-tube electrophoresis; Lipoprint LDL System (Quantimetrix, Redondo Beach, CA)	
Varady KA *et al.* [51]	60 obese subjects	↓	ADMF is an efficient strategy for decreasing of sdLDL level and increasing LDL particle size
		Non-denaturing 2%–16% polyacrylamide gradient gel electrophoresis	
Stanhope KL *et al.* [52]	32 overweight and obese subjects	↑	Dietary fructose promotes dyslipidaemia
		(with fructose) Precipitation	
Miyashita M *et al.* [53]	30 obese children	↑	High prevalence of sdLDL in obese children; a relationship of peak LDL diameter with abdominal fat accumulation, HDL-C and TG levels
		Gel electrophoresis	
King RF *et al.* [54]	65 overweight and obese children	↓	Reduction in LDL-C, LDL-C III and LDL-C peak particle size
		Ultracentrifugation	
Tascilar ME *et al.* [55]	26 obese children (13 girls, 13 boys)	↔	Measurement of LDL particle size is not necessary in childhood obesity
		Polyacrylamide gradient gel electrophoresis	
Maffeis C *et al.* [56]	10 obese boys	↑	A change of ≈25% of energy load from fat to carbohydrate in a meal improves postprandial pro-atherogenic factors in obese boys
		After the HF meal (abstract; no data for measuring)	
Makimura H *et al.* [58]	102 normal weight and obese men and women	↑	An independently association between reduced peak-stimulated GH and an atherogenic lipoprotein profile in obesity
		GH↓	
		GH stimulation testing and NMR spectroscopy	
Goedecke JH *et al.* [60]	15 normal-weight black; 15 normal-weight white; 13 obese black; 13 obese white South African women	↑	Obese black women had significantly more sdLDL compared to obese white women
		Non-denaturing 2 to 16% polyacrylamide gradient gel electrophoresis	
Filippatos TD *et al.* [63]	89 overweight and obese patients	↓	Orlistat + fenofibrate led to a greater reduction in sdLDL-C levels and favourable effects on Lp-PLA2
		3% polyacrylamide gel-tube electrophoresis; Lipoprint LDL System (Quantimetrix, Redondo Beach, CA)	
Nakou ES *et al.* [64]	86 overweight and obese patients with hypercholesterolemia	↓	The combination orlistat and ezetimibe have a more favourable effect on LDL-C and sdLDL-C levels than either drug alone
		3% polyacrylamide gel-tube electrophoresis; Lipoprint LDL System (Quantimetrix, Redondo Beach, CA)	

LDL-C—low density lipoprotein cholesterol; sdLDL—small dense LDL; HDL-C—high density lipoprotein cholesterol; TG—triglycerides; Lp-PLA2—lipoprotein-associated phospholipase A2; GH—growth hormone; ADMF—alternate day modified fasting, MetS—metabolic syndrome; HF—high fat; NMR—nuclear magnetic resonance; ↑: increased; ↓: decreased; ↔: no effect.

## 4. Treatment Options

Given that obese individuals with mixed dyslipidaemia have raised sdLDL-C levels, agents that have a potential beneficial effect on this LDL phenotype may be useful [65]. In visceral obesity, drugs, such as statins, fibrates and insulin sensitizers [66], are often required to correct any associated dyslipidaemia [67]; cannabinoid receptor type 1 blockers (such as rimonabant) are not currently on the market [52]. Furthermore, statins and other hypolipidemic agents (e.g., ezetimibe and fibrates), as well as weight-reducing agents were reported to favourably affect LDL subfractions [63,64]. Briefly, ezetimibe decreased the large and medium LDL particles and, to a lesser extent, the sdLDL particles, while it had no influence on LDL size; however, its sdLDL lowering capacity may be enhanced in individuals with elevated TG levels [65,68]. It seems that fenofibrate is equally or even more effective than statins in reducing sdLDL levels and increasing LDL size [69]. Fibrates and niacin reduced sdLDL levels and shifted LDL size towards large, buoyant LDL particles [70].

It was shown that a normalization of adiposity could lead to conversion from pattern B to pattern A LDL phenotype [71]; weight loss through energy restriction also increased LDL particle size and, thus, reduced CHD risk in obese subjects [72]. Several anti-obesity drugs were withdrawn from use over the past few years, due to adverse events, and currently, orlistat is available for long-term obesity management [66]. Orlistat significantly reduced both LDL-C and sdLDL-C levels (−19% and −45%, respectively, all *p* < 0.01); however, the decrease in sdLDL-C levels (−76%; *p* < 0.01 *vs.* baseline) and the increase in LDL particle diameter (+1.4%; *p* < 0.01 *vs.* baseline) were greater with the combination of ezetimibe and orlistat compared with either monotherapy (*p* < 0.05) [64]. These authors explained this greater fall in sdLDL-C levels by the greater decrease in TG in the combination group compared with monotherapy. In addition, orlistat, in both cases, alone or in combination with ezetimibe, improved anthropometric and metabolic variables (BMI, HOMA, serum uric acid, transaminase activities and plasma Lp-PLA2 [64]). In an earlier study, multiple regression analysis showed that the orlistat-induced reduction in sdLDL-C levels was significantly and independently correlated with the reduction in TG and HOMA [63]; orlistat raised sdLDL-C levels by 35% (*p* < 0.05 *vs.* baseline) and LDL particle diameter by 0.7% (non-significantly, *p* > 0.05). Furthermore, the combination treatment (orlistat + fenofibrate) was associated with decreases in VLDL-C (−36%; *p* < 0.01 *vs.* baseline) and sdLDL-C levels (−77%; *p* < 0.001 *vs.* baseline), as well as the proportion of the sdLDL-C of the total LDL-C (−68%; *p* < 0.01 *vs.* baseline) [63]. Orlistat + fenofibrate led to a greater reduction in sdLDL-C levels (*p* <0.05), together with a greater increase in LDL particle diameter (*p* < 0.05) compared with the orlistat group. Lp-PLA2 activity was also significantly decreased [63]. In addition, it was suggested that orlistat, alone or in combination with fenofibrate, may decrease LDL concentrations in obese MetS patients, confirming the findings that obese T2DM subjects with MetS orlistat + diet improved several CVD risk factors (fasting glucose, glycosylated haemoglobin (HbA1c), total cholesterol (TC) and LDL-C levels, systolic blood pressure (SBP), waist circumference and HOMA) compared with diet and exercise alone [73]. 

Bariatric surgery is an effective treatment option in young obese patients with BMI > 40 kg/m^2^ or BMI > 35 kg/m^2^ in the presence of significant comorbidities [74,75]. A gender-dependent relationship between excess weight loss (EWL) and lipid subfractions (reduced TC, LDL-C, TG and HOMA-IR; *p* < 0.0005 for all) after laparoscopic Roux-en-Y gastric bypass (LRYGB) has been reported [76]. Furthermore, a reduction of sdLDL with a rise in LDL relative flotation was observed after laparoscopic gastric banding (LAGB) (0.34 ± 0.04 *vs.* 0.38 ± 0.03; *p* < 0.001), but neither weight reduction nor changes in phospholipid fatty acid composition were found, despite a reduction in TG levels [77]. In general, LRYGB seems to be more effective, safer and has lower mortality rates compared with LAGB [78].

Although rimonabant has been withdrawn from the market, monotherapy with this drug led to a decreased sdLDL proportion [79,80], whereas in combination with ezetimibe or fenofibrate, a non-significant reduction in sdLDL was observed [65]. The effect of sibutramine, which has also been withdrawn from the market, on the sdLDL profile has not been described. Recently, a meta-analysis reported that orlistat and rimonabant could lead to an improvement in CVD risk factors (reduced SBP, diastolic BP, TC, LDL, fasting glucose, as well as body weight), whereas sibutramine might increase CVD risk factors (*i.e.*, hypertension); future studies should fully elucidate the effects of any similar drugs on cardiovascular risk factors, if they are marketed [81].

## 5. Effects of New Anti-Obesity Drugs on Lipids

In the field of anti-obesity drugs, the U.S. Food and Drugs Administration (FDA) has recently (June–July 2012) approved two new agents, namely lorcaserin (Belviq; a selective 5-hydroxytryptamine receptor 2c agonist) [82] and qsymia (formerly named qnexa, a combination of phentermine with controlled release topiramate) [83]. Of note, lorcaserin is contraindicated in combination with drugs for migraine and depression, as well as agents that can activate serotonin receptors or increase serotonin [82]. Patients with valve abnormalities should be carefully monitored, as serotonin receptors may induce valvular heart disease [84]. With regard to qsymia, heart rate should be monitored regularly during treatment, whereas patients with hyperthyroidism, glaucoma, recent (within the last six months) or unstable heart disease and stroke should not receive it [83]. Both drugs should be discontinued if <5% of initial body weight is lost within three months of treatment.

Contrave (naltrexone sustained-release (SR) combined with bupropion SR) was rejected by the FDA in 2011, due to concerns on CVD risk; however, the FDA agreed to consider the results of a currently running cardiovascular outcomes [85]. These anti-obesity drugs were shown not only to reduce weight, but also to beneficially affect the cardiometabolic profile of obese patients (e.g., waist circumference, lipids, fasting glucose and insulin sensitivity) [86,87,88]. With regard to lipids, these drugs were reported to significantly reduce TC, LDL and TG and increase HDL [86,88,89,90]. However, these data are scarce and, even more, no data exist regarding sdLDL. Future larger studies are needed to establish the effect of these anti-obesity drugs on lipids.

## 6. Effects of Lipid-Lowering and Anti-Obesity Drugs on HDL Subfractions

HDL-C also circulates in several subclasses with different properties, composition, metabolism and pathophysiological significance [91,92]. The HDL subfraction distribution may potentially affect the multiple actions of HDL with clinical consequences [93,94], but the role of each individual HDL subfraction on CVD risk remains inconclusive [95,96].

MetS has been linked to increased small HDL-3 particles and reduced large HDL-2 levels [97]. Patients with acute ischemic stroke were reported to have smaller HDL size with less HDL 2b and more HDL 3a, 3b and 3c subclasses [98].

Interestingly, atorvastatin was shown to increase HDL particle size in hyperlipidemic patients [99]. Similarly, rosuvastatin alone or in combination with ω-3 fatty acids raised larger HDL subfractions, whereas rosuvastatin with fenofibrate increased small HDL levels [100].

Gemfibrozil was found to increase small HDL subclasses in the Veterans Affairs High-Density Lipoprotein Intervention Trial (VA-HIT) [101]. In contrast, when combined with niacin, gemfibrozil raised large HDL-2 levels [102].

Niacin treatment was associated with increases in large HDL particles in both type 2 diabetics [103] and hyperlipidemic patients [102]. Interestingly, the beneficial effects on HDL particles (*i.e.*, increase in large HDL and reduction in small HDL) were numerically greater following niacin plus simvastatin combination therapy compared with atorvastatin monotherapy in a post-hoc analysis of 137 hyperlipidemic patients of the SUPREME study [104]. In another study, niacin monotherapy, as well as its combination with simvastatin raised both HDL-3 and HDL-2 levels, the latter in a relatively greater degree, in dyslipidemic individuals [105]. 

Pioglitazone and metformin were shown to beneficially affect both LDL and HDL subclasses in type 2 diabetic patients [106,107]. In contrast, rosiglitazone was found to increase smaller HDL and reduce larger HDL particles in patients with T2DM [108], as was troglitazone [109]. Of note, both rosiglitazone and troglitazone were withdrawn from the market, due to severe adverse effects (cardiovascular and hepatic disorders, respectively).

Cilostazol, a selective inhibitor of phosphodiesterase 3A used in patients with intermittent claudication to improve their walking distance, was also shown to beneficially affect HDL subclasses [110]. This may be relevant if obese patients have peripheral artery disease. 

In obese hyperlipidemic patients, orlistat and ezetimibe, alone or in combination, although not affecting HDL quantity, led to significant changes in HDL quality [111]. In detail, orlistat raised HDL-2 and decreased HDL-3 subclasses, whereas ezetimibe and their combination reduced HDL-3 subfraction. Similarly, in another study with obese MetS patients, orlistat increased large HDL and decreased small HDL subclasses, whereas fenofibrate raised small HDL particles [112]. Ezetimibe was also reported to reduce small HDL subfractions in patients with primary dyslipidaemia [113].

With regard to the new anti-obesity drugs (*i.e.*, lorcaserin, qsymia and contrave), no data exist on their impact on HDL subclasses; future studies are required to evaluate any possible effect.

Small HDL3c particles in MetS subjects failed to protect endothelial cells from oxLDL-induced apoptosis, and a negative correlation between HDL3c-mediated protection from apoptosis and waist circumference, plasma TG, TC/HDL-C ratio and oxLDL were found [114]. This deficiency in anti-apoptotic activity of small HDL was associated with altered apolipoprotein and lipid composition.

## 7. Lifestyle as the First-Line Therapeutic Option

Changes in the HDL profile were reported in overweight diabetic men and women following weight reduction [115]. Weight loss has been suggested as an effective method in reversing the decrease in HDL levels in obesity, and weight loss achieved through exercise is more effective than weight loss achieved by diet [116]. In a study with 46 obese subjects [117], only the combination of alternate day fasting plus exercise resulted in decreased LDL-C (*p* < 0.05) and increased HDL-C (*p* < 0.05) with a decreased proportion of small HDL particles (*p* < 0.01) compared with each intervention alone. Several recent meta-analyses [118,119,120] have also reported beneficial effects of exercise on lipids and lipoproteins in patients with MetS, in accordance with the results of many studies that have reported that exercise could have an important role in increasing HDL-C level [117,121,122]. Interestingly, when associations of all-cause mortality, adiposity and fitness were examined in older adults, fitness was inversely associated with mortality, and results were changed a little by adjustments for adiposity or fat distribution [123]. Thus, the authors have found that both fitness and BMI are independent predictors of all-cause mortality in adults 60 years old or older, emphasizing that further studies are needed to confirm if total adiposity *per se* may be the factor that increases mortality risk. The same authors have shown that lower levels of fitness are also associated with a higher risk of all-cause and CVD mortality in younger and middle-aged men [124,125].

In a six-month randomized study [126] performed in 78 severely obese subjects (86% of them had either diabetes or MetS), the authors found a favourable effect of a low carbohydrate diet on lipoprotein subfractions compared with a conventional diet, while both diets similarly decreased LDL particles and increased large HDL. In addition, both diets significantly decreased postprandial lipemia and led to similar (but non-significant) alterations in the total cholesterol/HDL-C ratio, fasting triacylglycerols, oxLDL and LDL subclasses distribution [127]. However, a very low carbohydrate diet prevents a decrease in HDL-C, although it did not lower LDL-C compared with a low-fat weight loss diet [127]. Similar results were also achieved when both diets were consumed for weight maintenance rather than weight loss, thereby reducing the effect of change in body weight on lipids [128]. In a study that compared the effects of pioglitazone *vs.* diet/exercise on body fat, glucose and lipid metabolism in obese, insulin-resistant individuals [129], only diet/exercise reduced total cholesterol, TG and LDL-C concentrations, whereas both treatment increased large LDL and decreased sdLDL particles. Furthermore, a Mediterranean-style diet rich in fruits and vegetables and with high polyunsaturated fats reduced cardiovascular events and decreased LDL-C with a concomitant increase in HDL-C [130].

Increased physical activity may improve sdLDL in hyperlipidemic subjects [131]. In general, lifestyle modification is the first-line therapy [132], with smoking cessation, exercise, mild-to-moderate alcohol consumption and weight loss increasing HDL-C in patients with levels <40 mg/dL [133]. In summary, first-line therapy in obese patients should be a healthy diet and physical activity. Lipid-lowering therapies should be prescribed only after or together with lifestyle modifications, unless the risk of a vascular event is so high that the prescribing clinician elects not to delay effective pharmacological intervention. If after statins therapy, HDL-C remains low, then niacin should be prescribed.

It is known that increased LDL-C (including decreased HDL-C) is one of several factors that are linked with high cardiovascular risk. However, the exact mechanisms by which LDL particle size may act as an independent CVD risk factor remains unclear. Currently, there is no strong clinical evidence to show that targeting lipoprotein subfractions is useful or recommended. Further large studies are needed to elucidate the clinical implications of lipoprotein subfractions, as well as to establish the effect of the here mentioned anti-obesity drugs on lipoproteins.

## 8. Conclusions

In overweight and obese hypercholesterolemic subjects, statins are the first treatment choice. Orlistat and ezetimibe coadministration had a more favourable effect on LDL-C and sdLDL-C levels than either drug alone [64]. This combination also exhibited favourable effects on Lp-PLA2 activity and LDL phenotype in overweight and obese patients with MetS, but positive effects on the LDL distribution profile should be replicated in larger studies. ADMF may increase LDL particle size, thus decreasing the sdLDL proportion, and ADMF could be a suitable alternative to traditional energy restriction for lowering CVD risk in obese individuals.

Anti-obesity medications, either as monotherapy or in combination with other drugs, presented encouraging results in terms of weight loss, safety and improvement of cardiometabolic risk factors. Combinations of drugs targeting different pathways in low doses may have better results than strategies that modify one pathway alone; this might represent the future strategy in obesity treatment. 

Improving the quantity and the quality of lipoproteins in obesity and the MetS may represent an effective approach to reduce cardiovascular risk.
